# Remimazolam Versus Propofol in General Anesthesia of Complex Surgery in Critical and Non-Critical Patients: Meta-Analysis of Randomized Trials

**DOI:** 10.3390/jcm13247791

**Published:** 2024-12-20

**Authors:** José Luis Muñoz-Carrillo, Natalie Rodríguez-Cortes, Sandra Trujillo Lévano, Cristian Moran-Mariños, Joshuan J. Barboza

**Affiliations:** 1 Laboratorio de Inmunología, Centro Universitario de los Lagos, Universidad de Guadalajara, Lagos de Moreno, Jalisco 47460, PC, Mexico; mcbjlmc@gmail.com; 2 Facultad de Medicina, Universidad El Bosque, Bogotá 110121, PC, Colombia; natalirodriguez@unbosque.edu.co; 3 Facultad de Medicina Humana, Universidad Científica del Sur, Lima 15842, PC, Peru; trujillosandra17@gmail.com; 4 Unidad de Investigación en Bibliometría, Vicerrectorado de Investigación, Universidad San Ignacio de Loyola, Lima 15024, PC, Peru; cp.moran94@gmail.com; 5 Escuela de Medicina, Universidad Señor de Sipán, Chiclayo 14001, PC, Peru

**Keywords:** remimazolam, propofol, general anesthesia, randomized controlled trial, systematic review, meta-analysis

## Abstract

**Objective**: To compare the efficacy and safety of remimazolam with propofol in general anesthesia in adult patients. **Methods**: A systematic search in Pubmed, Scopus, Web of Science, and Embase was performed. Patients undergoing complex surgery who were critically ill or non-critically ill were included. The risk of bias (RoB) 2.0 tool was applied. Random-effects models using the inverse variance method were applied for all meta-analyses. **Results**: Nine randomized controlled trials were included (patients taking remimazolam, n = 678; propofol, n = 454). Remimazolam compared to propofol is likely to produce a large decrease in intraoperative hypotension (RR 0.62, 95% CI 0.50 to 0.76, I2 = 63%, n = 9, CoE moderate certainty), incidence of respiratory depression (RR 0.28, 95% CI 0.09 to 0. 82, I2 = 0%, n = 3; CoE moderate certainty), injection site pain (RR 0.14, 95% CI 0.02 to 0.94, I2 = 21%, n = 4; CoE moderate certainty), and may produce little or no difference in bradycardia (RR 0.61, 95% CI 0.36 to 1.06, I2 = 0%, n = 4; CoE moderate certainty). **Conclusions**: In patients undergoing complex surgery who are critically ill or non-critically ill, remimazolam, compared with propofol, is likely to produce a large decrease in intraoperative hypotension, incidence of respiratory depression, and injection site pain, but little or no difference in bradycardia is possible.

## 1. Introduction

The efficacy and safety of anesthetic agents are critical considerations for general anesthesia procedures [1]. Remimazolam, a relatively new short-acting benzodiazepine that acts on the gamma-aminobutyric acid type A receptor (GABAAR), has been increasingly compared with propofol, a widely used agent, for its anesthetic properties and patient safety outcomes [2]. Remimazolam has been shown to provide adequate anesthesia with rapid induction and recovery times that are comparable to those of propofol. A study by Shimizu et al. found that remimazolam led to rapid recovery following anesthesia, although it might cause delayed psychomotor decline compared to propofol [3]. Additionally, Song et al. reported that patients administered remimazolam for anesthesia maintenance experienced a minor decrement in quality of recovery compared to those who received inhalant anesthetic agents like desflurane [4]. Furthermore, remimazolam safety is noteworthy in patients with severe cardiovascular conditions. Studies like Kitaura et al. have indicated that remimazolam causes less cardiovascular depression, making it a safer choice for patients with advanced heart failure or those undergoing complex cardiovascular procedures [5]. However, more is needed to help further clarify the possible benefits and limitations of remimazolam compared to traditional anesthetics such as propofol. For this reason, this systematic review aims to compare the efficacy and safety of remimazolam with propofol in general anesthesia in adult patients.

## 2. Materials and Methods

### 2.1. Study Design

This study involved a systematic review and meta-analysis guided by the Preferred Reporting Items for Systematic Reviews and Meta-Analyses (PRISMA-2020) standards.

### 2.2. Searches

We used databases such as PubMed, Scopus, Web of Science, and EMBASE for our searches. These searches covered the database from its inception until 31 May 2024 and employed critical phrases, MeSH terms (for PubMed), and Emtree thesauri (for Scopus and EMBASE). A specific search strategy was applied to each database (Supplementary Table S1), with primary search phrases including (“Remimazolam”) AND (“Propofol”) AND (“General anesthesia”) AND (“Randomized controlled trial”). There were no restrictions on language or publication date. Additionally, reference lists of relevant studies and included review articles were manually searched for other potentially eligible trials.

### 2.3. Eligibility Criteria

This study included all studies that meet the following criteria: randomized controlled trials, phase II or III, involving a population (P) of adults aged 18 or older undergoing general anesthesia (induction and maintenance) and undergoing complex surgery who are critically ill or non-critically ill (emergency surgery, spinal surgery, endovascular and heart surgery, orthopedic surgery). Excluded were cosmetic surgeries, gastroscopies, and outpatient surgeries, among others. Intervention (I): Induction and maintenance dosing with remimazolam and control (C): propofol (no restrictions on dosage or method of administration), and American Society of Anesthesiologists (ASA) Physical Status Classification III or lower. The studies must compare the efficacy and safety of remimazolam as an intervention to propofol for general anesthesia.

Exclusion criteria: studies using other anesthetic drugs in combination with remimazolam and propofol, conference abstracts, systematic reviews, narrative reviews, case reports, series, cohort studies, and letters to the editor will be excluded.

### 2.4. Outcomes

The primary outcomes were the incidence of intraoperative hypotension and the incidence of respiratory depression (measured through frequencies and relative risk). The secondary outcomes were bradycardia and injection site pain (measured through the occurrence rates of these adverse events).

### 2.5. Data Extraction

Following the electronic searches, the results were gathered into a single library, where duplicates were removed. The initial screening was conducted by evaluating the titles and abstracts and applying the inclusion and exclusion criteria to each reviewed result using the Rayyan platform. Studies past this phase were retrieved and analyzed in full text, followed by another screening process to justify the inclusion and exclusion criteria. After this step, the eligible studies were included in the systematic review, and data extraction was started. A third review author (JJB) was consulted in case of any disagreements.

A pre-formatted Microsoft Excel spreadsheet was used to extract data, ensuring that data from each study were collected individually and blinded. Information was gathered for each survey of the author, year of publication, country, study type, number of participants per intervention arm, selection criteria, description of the intervention and control, and primary and secondary outcomes.

### 2.6. Risk of Bias Assessment

The risk of bias (RoB) was independently evaluated using the RoB 2.0 tool (University of Bristol © 2024). Any discrepancies in the assessment were resolved by consulting a third author (JJB). The RoB for each domain and study was classified as low, with some concerns, or high for randomized controlled trials (RCTs).

### 2.7. Data Synthesis

Random-effects models using the inverse variance method were applied for all meta-analyses comparing the effects of remimazolam with propofol on primary and secondary outcomes (6). The between-study Tau2 variance was calculated using the Paule–Mandel method. If more than five studies are included in the meta-analysis, the Hartung–Knapp adjustment will be utilized (7).

The impact of remimazolam versus propofol on dichotomous outcomes was presented as relative risks (RRs) with 95% confidence intervals (CIs). Null events in one or two arms of the randomized controlled trials (RCTs) were adjusted using the continuity correction method.

Statistical heterogeneity among the RCTs was evaluated using the I2 statistic, which indicates low (<30%), moderate (30–60%), and high (>60%) levels of heterogeneity.

Fixed effects models and the Mantel–Haenszel method were employed for sensitivity analysis. The metabin function from the R 3.5.1 meta library (www.r-project.org) was used. To assess publication bias, both a funnel plot and Egger’s test were conducted.

GRADE assessment: The certainty of the evidence and the degree of recommendation of the intervention were assessed using the GRADE methodology (8). This assessment evaluated various domains, such as risk of bias, inconsistency, indirectness, imprecision, and publication bias. The certainty of the evidence was determined based on the outcomes and described in the summary of findings (SoF) tables, which were created using the online software GRADEpro GDT (Copyright © 2021, McMaster University and Evidence Prime Inc., Hamilton, ON, Canada) (Supplementary Table S2).

## 3. Results

### 3.1. Selection of Studies

**Figure 1:**
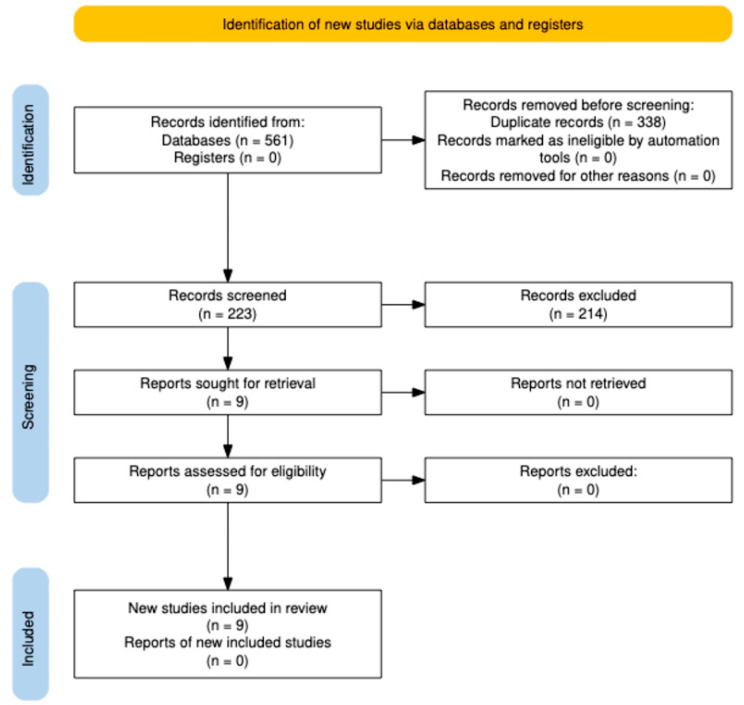
Flow chart of selection studies.

Figure 1 shows the PRISMA flow diagram of the identification, screening, exclusion, and inclusion of studies carried out in this systematic review and meta-analysis. A total of 561 records were obtained from the PubMed (n = 121), Scopus (n = 191), Web of Science (n = 89), and Embase (n = 160) databases; 338 record duplicates were removed, and 224 reports were excluded using automatic filters or manually after selecting titles and abstracts, and reports were assessed for eligibility. Finally, nine randomized controlled trials were included in the systematic review [2,4,6,7,8,9,10,11,12].

### 3.2. Characteristics of Included Studies

The systematic review below covers a few studies conducted to evaluate the efficacy and safety of remimazolam compared with propofol in various surgical settings. We have provided complete descriptions of the included studies, including design, population, interventions, comparators, and outcomes. Regarding the features of designs, there were nine randomized controlled trials and prospective, double- [2,4,7,8,9,10,11,12] and single-blind [6], phase IIb/III [6,7], and phase III [2,4,8,9,10,11,12] trials.

There were 1132 patients assessed (remimazolam = 678; propofol = 454); in the remimazolam group, the average age of the patients was 55.5 years (SD: 6.5), with an average weight of 63.3 kg (SD: 3.0) and an average body mass index (BMI) of 24.4 (SD: 1.5). In contrast, the patients in the propofol group had an average age of 55.1 years (SD: 6.8), an average weight of 64.6 kg (SD: 2.2), and an average BMI of 24.3 (SD: 1.1) (Table 1).

All the included studies were randomized controlled trials (RCTs) that investigated the efficacy and safety of remimazolam compared to propofol as an anesthetic agent. In these trials, remimazolam was used as the intervention drug, with induction doses ranging from 0.2 mg/kg to 12 mg/kg/h and maintenance doses typically between 1 mg/kg/h and 20 mg/kg/h. Propofol served as the control drug, with induction doses of 1–2.5 mg/kg and maintenance doses ranging from 4 to 12 mg/kg/h, often administered through a target-controlled infusion (TCI) model. The outcomes reported varied across studies but generally included the incidence of intraoperative complications, such as hypotension, the time to emergence from anesthesia, and quality of recovery scores (e.g., QoR-15 score).

Secondary outcomes frequently assessed included hemodynamic parameters like blood pressure and heart rate, adverse events such as bradycardia or postoperative nausea and vomiting, and the depth of anesthesia, monitored using the Bispectral Index (BIS). Continuous monitoring of vital signs, including mean arterial pressure (MAP), heart rate (HR), oxygen saturation (SpO_2_), and BIS, was a consistent feature across all studies. Notably, Shi et al., 2022, Doi et al., 2020, and Song et al., 2023 [4,9,10] used flumazenil to expedite recovery in the remimazolam group if necessary. Overall, these studies aimed to compare the two anesthetics to determine which offered better hemodynamic stability, safety, and quality of recovery for patients undergoing surgery (Table 2).

### 3.3. Risk of Bias Assessment

**Figure 2:**
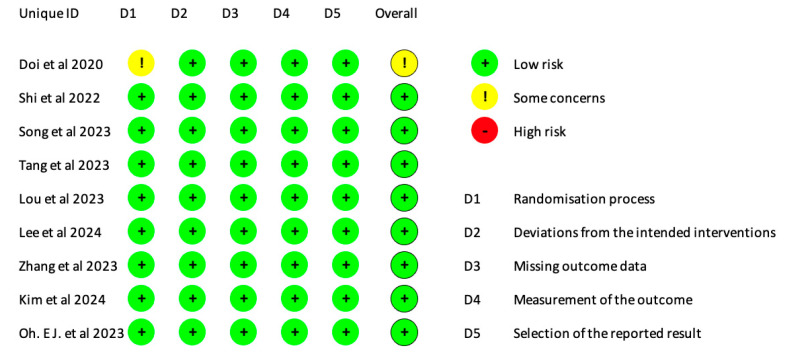
Risk of bias assessment.

One study had some concerns about risk bias in domain 1 (randomization process) (Doi et al. (2020) [9]). All studies had a low risk of bias (Figure 2).

### 3.4. Effect of Remimazolam Versus Propofol in the Primary and Secondary Outcomes

**Figure 3:**
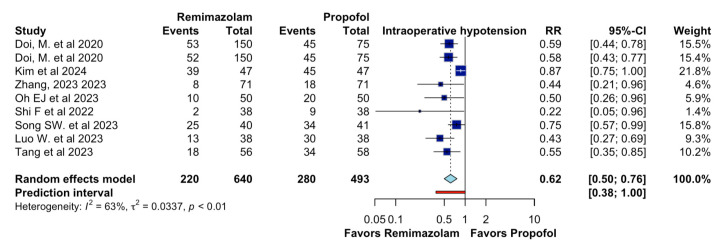
Effects of remimazolam on intraoperative hypotension.

In adults undergoing general anesthesia, remimazolam, compared to propofol, likely results in a large decrease in intraoperative hypotension (RR 0.62, 95%CI 0.50 to 0.76, I2 = 63%, n = 9; CoE moderate certainty; Figure 3).

**Figure 4:**
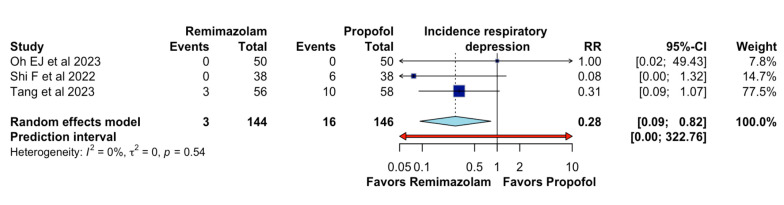
Effects of remimazolam on incidence of respiratory depression.

In adults undergoing general anesthesia, remimazolam, compared to propofol, likely results in a large decrease in the incidence of respiratory depression (RR 0.28, 95%CI 0.09 to 0.82, I2 = 0%, n = 3; CoE moderate certainty; Figure 4).

**Figure 5:**
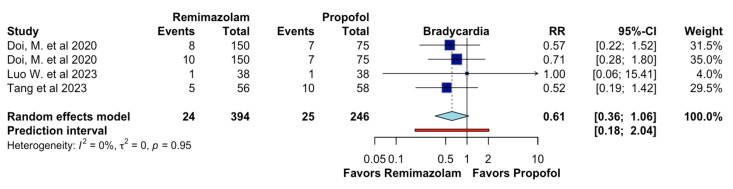
Effects of remimazolam on bradycardia.

In adults undergoing general anesthesia, remimazolam, compared to propofol, may result in little to no difference in bradycardia (RR 0.61, 95%CI 0.36 to 1.06, I2 = 0%, n = 4; CoE moderate certainty; Figure 5).

**Figure 6:**
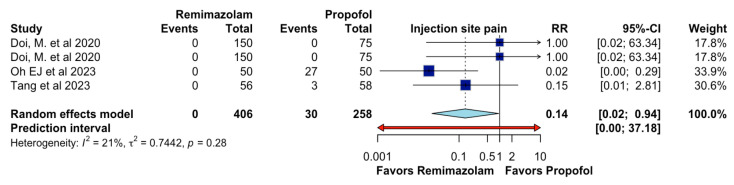
Effects of remimazolam on injection site pain.

In adults undergoing general anesthesia, remimazolam, compared to propofol, likely results in a large decrease in injection site pain (RR 0.14, 95%CI 0.02 to 0.94, I2 = 21%, n = 4; CoE moderate certainty; Figure 6).

## 4. Discussion

This meta-analysis illustrates that, on certain critical parameters, remimazolam outperforms propofol during general anesthesia. Specifically, remimazolam has a significantly lowered incidence of intraoperative hypotension—as suggested by the RR of 0.62 and 95% CI of 0.50–0.76—and respiratory depression—as suggested by the RR of 0.28 and 95% CI of 0.09–0.82—indicating greater hemodynamic and respiratory stability. Additionally, although the gap in the incidence of bradycardia between the two agents was narrower, remimazolam had a clinically significant advantage in reducing the incidence of injection site pain, with a risk ratio of 0.14 and 95% CI of 0.02–0.94, further enhancing the patient experience of anesthetic administration. These results suggest that remimazolam is likely to provide substantial clinical benefits over propofol for the anesthetic management of adult patients.

The moderate certainty of evidence supporting remimazolam over propofol for the reduction of intraoperative hypotension and respiratory depression suggests these results could be reliable but that future studies might differ from these estimates. In this sense, Yang et al. found that remimazolam reduced intraoperative hypotension (RR 0.58, 95% CI 0.47 to 0.71). Together, these findings show the advantages of remimazolam in patients undergoing general anesthesia, particularly in those with cardiovascular risks [13].

Although intraoperative and postoperative hypotension have been identified as major adverse problems and a target for research and interventions, BP changes during and after procedural sedation have been less well documented, and their significance, if any, has not been determined [14].

Anesthesiologists, and increasingly non-anesthesiologists, widely use propofol for procedural sedation; however, the use of remimazolam is supported based on findings and evidence [15].

This meta-analysis also shows remimazolam reduces respiratory depression. In this respect, the study by Zhu et al. [16] found a reduction in respiratory depression between the use of remimazolam (n = 39/627; 6.2%) vs. propofol (n = 75/628; 11.9%; *p* < 0.001).

Safety in terms of induction or maintenance with the use of anesthesia is a relevant aspect to consider, as although sedation is generally considered safe in most patients, adults undergoing sedation are associated with increased risk of hemodynamic instability, respiratory depression, and delayed discharge, especially in those with cardiopulmonary disease [17].

However, for bradycardia, there was no significant difference between remimazolam and propofol. Our meta-analysis shows the reduction in bradycardia was not substantial, which lines up with other reviews showing minimal difference between these two agents (1, 4, 5). So, while remimazolam has strong hemodynamic and safety benefits, its impact on bradycardia is not so significant compared to propofol. Zhang et al. found that the incidence of bradycardia in the remimazolam group was lower than in propofol (n = 0/41, 0.0% vs. 1/41, 2.4%), although these differences were not significant (*p* = 0.314) [18].

Remimazolam has big clinical benefits over propofol in general anesthesia, especially in high-risk groups like elderly patients, people with heart problems, and those having complex surgeries. It has superior hemodynamic stability, which reduces the need for vasopressors during surgery, which is super important in cardiac and neurovascular procedures [19].

Also, remimazolam lowers pain at the injection site and does not cause much bradycardia, making the overall anesthesia experience more comfortable [20]. Its predictable safety profile and stable effects support its use in high-risk surgical settings where control of blood pressure and breathing is critical [21].

This study has some limitations. Regarding the generality of surgery, our study classified complex surgery according to the type of patient (American Society of Anesthesiologists (ASA) Physical Status Classification III or lower), so there is an implicit heterogeneity between the types of patients included in this study.

This variability in surgical complexity may affect the comparability of outcomes in terms of the effects of different anesthetic interventions [22]. Patients undergoing less invasive surgeries may have faster recovery times and a lower risk of complications, which may not be comparable with the results obtained in highly complex surgeries involving a higher risk of perioperative complications and prolonged recovery times [23]. In addition, the different risks associated with each type of surgery (e.g., respiratory complications in cirrhotic patients) may limit the generalizability of the findings [24].

Finally, anesthetic demands vary significantly by type of surgery, which may influence the efficacy and safety of the anesthetic agents compared, making the results difficult to extrapolate to other patient populations or types of surgery. This limitation could impact the overall interpretation of the effectiveness of the interventions evaluated in the systematic review.

## 5. Conclusions

Remimazolam, compared to propofol, likely results in a large decrease in intraoperative hypotension, incidence of respiratory depression, and injection site pain but may result in little to no difference in bradycardia.

## Figures and Tables

**Table 1:** Characteristics of included studies.

Author, Year	Country	Type and Phase	NCT (Coding del Art)	N° Patients per Arm	Eligibility Criteria	Age (Mean, SD)	Weight (Mean, SD)
Doi, M. et al., 2020 [9]	Japan	Multicenter, single-blind, randomized, parallel- group, phase IIb/III trial	JapicCTI number: 121973	R1: 150	≥20 years old body weight of 100 kg or less scheduled for elective surgery ASA I–II. Exclusion Criteria: Emergency surgeries, extracorporeal circulation, spinal/epidural anesthesia, hepatectomy, liver transplant, uncontrolled hypertension, renal (creatinine ≥ 2 mg/dL) or hepatic (AST/ALT ≥ 2.5 × ULN) impairment.	R1: 57.7 (14.7)	W: R1: 61.1 (10) BMI: R1: 23.5 (3),
				R2: 150		R2: 56.2 (16)	R2: 60.5 (11.6), BMI: R2: 23 (3.1),
				P1: 75		P1: 56.3 (17.6)	P1: 60.8 (10.8), BMI: P: 23.3 (3.4)
Kim, 2024 [13]	South Korea	Prospective, randomized, controlled clinical trial	NCT05644483	R: 47	Eligibility Criteria: Included patients aged 19–80 years, ASA physical status 1–3, and undergoing major spinal surgery in the prone position. Excluded those with uncontrolled hypertension, significant cardiovascular/liver disease, glaucoma, alcoholism, or BMI < 15 or >35 kg/m^2^.	R: 67.4 ± 8.0	R: 61.1 ± 9.0 kg
				P: 47		P:67.2 ± 7.5	P:64.9 ± 9.2 kg
Zhang, 2023 [14]	China	Prospective, double-blind, randomized controlled, non-inferiority trial	NCT04950621	R: 71	Adults (18+ years) scheduled for cerebral endovascular procedures under general anesthesia. ASA grade IV or higher. Hunt-Hess grade III or higher. BMI < 18 kg/m^2^ or >30 kg/m^2^. Previous cerebral endovascular procedures or surgical clipping.	R:56.6 ± 10.2	R:24.2 ± 2.8 BMI
				P:71 (69)		P: 56.0 ± 9.9	P:24.5 ± 2.6 BMI
Lee 2024 [15]	South Korea	Prospective, double-blind, randomized controlled trial	NCT04994704	R:37	Inclusion Criteria: Individuals aged 20–70 years, ASA I or III, who are undergoing elective spine surgery with intraoperative neurophysiological monitoring. Exclusion Criteria: Addiction or dependence on alcohol or psychotropic substances. Hypersensitivity or tolerance to benzodiazepines. Body mass index (BMI) exceeding 30 kg/m^2^.	R: 54.2 (Range: 27–66)	R: 65.0 (9.4)
				P: 36		P: 50.3 (Range: 33–66)	P:65.5 (11.6)
Oh EJ, 2023 [12]	South Korea	Prospective, double-blind, randomized trial	KCT 0007132	R: 50	Eligibility Criteria: Patients between 19 and 75 years old ASA: I–III Reason for surgery: catheter ablation or atrial arrhythmia Exclusion Criteria: Adverse drug reactions (ADRs), moderate hepatic dysfunction, glaucoma, or metabolic disease.	R: 60 (Range: 54, 65)	BMI R: 26 (24.1, 27.7)
				P: 50		P: 60 (Range: 52, 64)	BMI P: 24.7 (22.5, 27.5)
Shi F, 2022 [10]	China	Randomized, single-blind, prospective, single-center controlled trial	ChiCTR2100045710	R: 38	Eligibility Criteria: Included patients aged 20–80 years scheduled for EVL with recent bleeding history. Excluded those with ASA IV/V, active variceal bleeding, recent alcohol use, hepatic encephalopathy, neurological diseases, hemorrhagic shock, or allergies to benzodiazepine/propofol.	R: 52.74 (4.93)	R: 68.26 (5.11)
				P: 38		P: 51.71 (5.48)	P: 66 (5.27)
Song SW, 2023 [4]	South Korea	Randomized parallel-group, single-blind controlled trial	KCT0007488	R: 40	Eligibility Criteria: Included patients aged 19–65 years with routine ACEI/ARB use and ASA status III or lower. Excluded emergency surgeries, BMI ≥ 35, uncontrolled hypertension, pregnancy, hepatic dysfunction (Class C), and those unable to consent.	R: 58.6 (6.4)	BMI R: 26.6 (4)
				P: 41		P: 60.1 (5.2)	BMI P: 26.3 (3.3)
Luo W, 2023 [11]	China	Single-center, randomized, placebo-controlled, blinded, parallel trial	ChiCTR2100048904	R: 39	Eligibility Criteria: Included adults aged 18–75 years, ASA I–II, BMI 18-<30 kg/m^2^, undergoing day surgery with LMA. Excluded those with benzodiazepine allergy, recent benzodiazepine/opioid use, alcohol abuse, significant renal/hepatic dysfunction, cardiorespiratory instability, recent drug trial participation, or pregnancy.	R: 43.5 (15.6)	BMI R: 22.7 (3.4) W R: 63.6 (12.6)
				P: 38		P: 44.3 (18.1)	BMI P: 23.2 (3,7) W P: 65.9 (13.5)
Tang, 2023 [2]	China	ECA, randomly allocated into the remimazolam group (R group) or the propofol group (P group) at a ratio of 1:1	ChiCTR2100053014	R: 56	Eligibility Criteria: Included patients aged 18–65 years, ASA I–II, undergoing arthroscopic meniscus repair. Excluded severe respiratory/circulatory diseases, chronic analgesic/sedative use, alcohol abuse, and inability to understand scales or self-care pre-surgery.	R: 48.5 (Range: 19–62)	BMI: R: 24.7 (2.93)
				P:58		P: 50 (Range: 19–64)	BMI P: 23.9 (2.85)

**Table 2:** Features of intervention and control of included studies.

Author, Year	INTERVENTION			CONTROL	OUTCOME
	Drug	Doses	Description	Description	
Doi, M. et al., 2020 [9]	Remimazolam	Remimazolan—Induction: 6 mg/kg/h or 12 mg/kg/h Remimazolan—Maintenance: 1.0–2.0 mg/kg/h	Induction: Administered at either 6 mg/kg/h or 12 mg/kg/h via continuous intravenous (IV) infusion until loss of consciousness (LoC), typically for up to 2.5 min. If LoC did not occur, the infusion was stopped, and another sedative was used. Maintenance: After induction, remimazolam was maintained at 1 mg/kg/h, adjustable up to a maximum of 2 mg/kg/h, depending on the patient’s condition, until the end of the surgery. Recovery: In cases where awakening was delayed, flumazenil was administered to expedite recovery.	Propofol—Induction: 2.0–2.5 mg/kg Propofol—Maintenance: 4–10 mg/kg/h Induction: Administered as a slow bolus of 2.0–2.5 mg/kg until LoC, which was expected to occur within 1 min. Maintenance: Continued infusion at 4–10 mg/kg/h, adjustable based on the patient’s condition until the end of surgery. Recovery: Patients were monitored for spontaneous recovery or given additional medication as needed.	Primary outcomes: (1) intraoperative awakening or recall, (2) the need for rescue sedative medication, and (3) body movement Secondary outcomes: (1) responding to verbal stimuli, (2) adequate recovery of respiratory function, (3) stable blood pressure (BP) and heart rate (HR), and (4) recovery of muscle strength
Kim, 2024 [13]	Remimazolam	Induction: 6 mg/kg/h. Maintenance: 1–2 mg/kg/h.	Induction with remimazolam was performed at a rate of 6 mg/kg/h. For anesthesia maintenance, the Remimazolam infusion was adjusted between 1–2 mg/kg/h to maintain a patient state index between 25 and 50	Propofol—Induction: Target-controlled infusion (TCI) using a Schneider pharmacokinetic model, with an effect-site concentration of 2.0 µg/mL and maintenance of 2–3 µg/mL. The propofol infusion rate was increased by 0.5 µg/mL every 30 s until loss of consciousness.	Primary outcomes: incidence of hypotensive episodes during the first hour after prone positioning Secondary outcomes included the incidence of severe hypotension and the total amount of inotropic or vasopressor medication
Zhang, 2023 [14]	Remimazolam	Induction: IV injection remimazolam 0.1 mg kg^−1^. Maintained with remimazolam 0.3–0.7 mg kg^−1^ h^−1^	After loss of consciousness upon anesthesia induction, Sufentanil (0.2–0.3 mg kg^−1^) and rocuronium (0.6–0.9 mg kg^−1^) were given to facilitate endotracheal intubation. Remifentanil was infused intravenously at 0.1–0.3 μg kg^−1^ h^−1^ during anesthesia maintenance. Rocuronium was injected intermittently as needed.	Induced with intravenous injection of propofol 1–1.5 mg kg^−1^ and maintained with propofol 4–10 mg kg^−1^ h^−1^. Also received Sufentanil and rocuronium for intubation and remifentanil for maintenance, with mechanical ventilation to maintain a Bispectral Index (BIS) between 40 and 60.	The primary outcome was time to emergence, defined as the interval from discontinuation of study drugs to eye opening upon verbal command (mITT): 16.1 ± 10.4 min in the remimazolam group vs. 19.0 ± 11.2 min in the propofol group. Secondary outcomes included the time to loss of consciousness (LoC); episode of hypotension (remimazolam group had lower rate of hypotensive episodes during anesthesia induction (11.3% vs. 25.4% in the propofol group, RR = 0.44 [95% CI: 0.21, 0.96], P = 0.03)); intraoperative use of vasoactive drugs; incidence of postoperative delirium (POD) during emergence; major complications prior to patient discharge; length of hospital stay and Glasgow Coma Scale (GCS) at discharge; the level of lactate in arterial blood, serum IL-6, TNF-α, and S100β at the end of the surgery; mRS at 30 and 90 days after surgery.
Lee 2024 [15]	Remimazolam	Remimazolam—Induction: 6–12 mg/kg/h Remimazolam—Maintenance 1–2 mg/kg/h	Induction with remimazolam at a rate of 6–12 mg/kg/h until loss of consciousness, followed by maintenance at 1.0–2.0 mg/kg/h. Concurrent administration of remifentanil using target-controlled infusion (TCI) based on the Minto model.	Propofol—Induction: 3.0 ng/mL^−1^. Induction using propofol at a targeted concentration of 3.0 ng/mL, followed by maintenance using the same target-controlled infusion method. Remifentanil was also administered similarly to the remimazolam group.	The primary outcome of our study was the total QoR-15 (Quality of Recovery) score measured on postoperative day (POD) 1. Secondary outcomes: We investigated hemodynamic parameters such as MAP, heart rate, peripheral oxygen saturation, and PSI values.
Oh EJ, 2023 [12]	Remimazolam	Remimazolan–Induction: 6 mg/kg/h Remimazolan–Maintenance: 1.0 y 2.0 mg/kg/h	Remimazolam Group: Patients received continuous infusion of remimazolam at 6 mg/kg/h for induction, adjusted to 1.0–2.0 mg/kg/h for maintenance. Maintenance: In both groups, remifentanil was infused at a rate of 0.05–0.20 μg/kg/min for analgesia. Rocuronium was used to achieve neuromuscular blockade.	Propofol Group: Patients received target-controlled infusion (TCI) of propofol at an effect-site concentration of 5.0 μg/mL for induction, adjusted to 3.0–5.0 μg/mL for maintenance. Maintenance: In both groups, remifentanil was infused at a rate of 0.05–0.20 μg/kg/min for analgesia. Rocuronium was used to achieve neuromuscular blockade.	Outcomes assessed Primary: obeying verbal commands Secondary: -Bispectral Index (BIS) -Time until laryngeal mask airway removal -RASS -Adverse events
Shi F, 2022 [10]	Remimazolam	Remimazolan–Induction: 0.2 mg/kg Remimazolan–Maintenance: 1 mg/kg/h	Induction: Remimazolam tosylate was administered as a slow bolus of 0.2 mg/kg. Maintenance: Following induction, remimazolam was continuously infused at a rate of 1.0–2.0 mg/kg/h during the procedure. Anesthesia Monitoring: All patients were monitored for vital signs, including mean arterial blood pressure (MAP), heart rate (HR), pulse oxygen saturation (SpO2), end-tidal CO2 partial pressure (PetCO2), and Bispectral Index (BIS). The depth of anesthesia was assessed using the Modified Observer’s Assessment of Alertness/Sedation (MOAA/S) scale. Recovery: At the end of the surgery, flumazenil was administered to patients in the remimazolam group to reverse the effects of the anesthetic and facilitate faster recovery. The same volume of saline was given to patients in the propofol group.	Propofol Group (Group P): Induction: Propofol was administered at 2 mg/kg. Maintenance: Continuous infusion of propofol was maintained at a rate of 4–10 mg/kg/h. Anesthesia Monitoring: All patients were monitored for vital signs, including mean arterial blood pressure (MAP), heart rate (HR), pulse oxygen saturation (SpO2), end-tidal CO2 partial pressure (PetCO2), and Bispectral Index (BIS). The depth of anesthesia was assessed using the Modified Observer’s Assessment of Alertness/Sedation (MOAA/S) scale.	Outcomes assessed Primary: success of the surgical procedure/MOAA Secondary: -Anesthetic time -Vital signs: MAP, heart rate -Hypotension -Adverse events after surgery: Hypotension, nausea and vomiting -Patient and operator satisfaction
Song SW, 2023 [4]	Remimazolam	Remimazolan–Induction: 6 mg/kg/h Remimazolan–Maintenance: 1 mg/kg/h	Remimazolam Group (Group R): Patients received a saline placebo followed by remimazolam at 6 mg/kg/h for induction and 1 mg/kg/h for maintenance. The infusion rate of remimazolam was adjusted if the Bispectral Index (BIS) exceeded 60. Maintenance: In both groups, remifentanil was infused at 0.25 µg/kg/min. Rocuronium was used for neuromuscular blockade, and orotracheal intubation was performed after two and a half minutes.	Propofol Group (Group P): Patients received propofol at 2 mg/kg for induction, followed by an infusion of remimazolam at 0.1 mg/kg/h for blinding purposes. Maintenance: In both groups, remifentanil was infused at 0.25 µg/kg/min. Rocuronium was used for neuromuscular blockade, and orotracheal intubation was performed after two and a half minutes.	Primary outcome was the incidence of hypotension following anesthesia induction. Secondary outcomes were heart rate, mean, systolic, and diastolic blood pressure (MBP, SBP, and DBP), and Bispectral Index (BIS).
Luo W, 2023 [11]	Remimazolam	Remimazolan—Induction: 0.3 mg/kg, Remimazolan—Maintenance: 1–3 mg/kg/h	RT Group: Received remimazolam tosylate at 0.3 mg/kg intravenously. If loss of consciousness (LoC) did not occur within 3 min, an additional dose of 0.1 mg/kg was administered. RT Group: Anesthesia was maintained with remimazolam tosylate at 1–3 mg/kg/h. Additional Medications: Sufentanil (0.2–0.4 µg/kg) was administered during induction, and rocuronium (0.2–0.4 mg/kg) was used for muscle paralysis before inserting a laryngeal mask airway (LMA). Monitoring and Adjustments: The depth of anesthesia was monitored using the Bispectral Index (BIS). Hypotension (SBP < 80% of baseline) was treated with ephedrine or phenylephrine, and sinus bradycardia (HR < 40 beats/min) was treated with atropine.	Induction: Patients in the propofol group received an intravenous bolus of propofol at a dose of 2.0–2.5 mg/kg. If loss of consciousness (LoC) did not occur within 3 min, an additional dose of 1.0 mg/kg of propofol was administered. Maintenance: After induction, anesthesia was maintained with a continuous infusion of propofol at a rate of 6–12 mg/kg/h. Remifentanil was concurrently administered at a rate of 0.05–0.15 µg/kg/min to provide analgesia during surgery. Monitoring: The depth of anesthesia was monitored using the Bispectral Index (BIS) to ensure it stayed within the desired range (typically between 40 and 60). Vital signs, including systolic blood pressure (SBP), diastolic blood pressure (DBP), heart rate (HR), and oxygen saturation (SpO2), were continuously monitored.	Outcomes assessed Primary: -Induction time -Alert time Secondary: -Success rate -BIS -MOAA -Adverse events (hypotension, bradycardia, hypoxia)
Tang, 2023 [2]	Remimazolam	Remimazolam–Induction: 6 mg/kg/h Remimazolam–Maintenance: 0.4–2 mg/kg/h	Induction: Patients in the R Group received remimazolam besylate at an intravenous infusion rate of 6 mg/kg/h for the induction of anesthesia. Maintenance: After induction, anesthesia was maintained by adjusting the remimazolam dose within a range of 0.4–2 mg/kg/h to achieve the desired depth of anesthesia, monitored using the Bispectral Index (BIS), which was targeted between 40 and 60. Remifentanil was also administered at a rate of 0.1–0.3 µg/kg/min to provide analgesia during the surgery. Procedure: Following the loss of consciousness (LOC), cisatracurium (0.2 mg/kg) was administered to facilitate the placement of a laryngeal mask airway (LMA). Volume-controlled mechanical ventilation was adjusted to maintain an end-tidal CO2 (EtCO2) concentration between 4.66 and 5.99 kPa.	Induction: Patients in the Propofol Group received propofol through plasma target-controlled infusion (TCI). The initial target concentration was set at 2 µg/mL and could be increased up to 3.5 µg/mL to achieve the desired level of anesthesia. Maintenance: After induction, anesthesia was maintained by adjusting the propofol concentration within the range of 1–3 µg/mL to maintain a Bispectral Index (BIS) between 40 and 60, ensuring appropriate depth of anesthesia. Remifentanil was administered at a rate of 0.1–0.3 µg/kg/min to provide analgesia during surgery. Procedure: Once loss of consciousness (LOC) was achieved, cisatracurium (0.2 mg/kg) was administered to facilitate the placement of a laryngeal mask airway (LMA). Volume-controlled mechanical ventilation was adjusted to maintain an end-tidal CO2 (EtCO2) concentration between 4.66 and 5.99 kPa.	Primary outcome: QoR-15 score Secondary outcomes: LOC BIS 60 Extubation time RSS Adverse events

## Data Availability

Data are contained within the article.

## Supplementary Materials

The following supporting information can be downloaded at: https://www.mdpi.com/article/10.3390/jcm13247791/s1, Supplementary Table S1. Strategy search; Supplementary Table S2. GRADE assessment.

## References

[1] Lee M., Lee C., Choi G.J., Kang H. (2024). Remimazolam for Procedural Sedation in Older Patients: A Systematic Review and Meta-Analysis with Trial Sequential Analysis. J. Pers. Med..

[2] Tang L., Sun Y., Hao X., Sun X., Xie C., Wang T., Hu C., Lu Y., Liu X. (2023). Effect of general anaesthesia with remimazolam versus propofol on postoperative quality of recovery in patients undergoing ambulatory arthroscopic meniscus repair: A randomised clinical trial. BJA Open.

[3] Shimizu T., Takasusuki T., Yamaguchi S. (2023). Remimazolam Compared to Propofol for Total Intravenous Anesthesia with Remifentanil on the Recovery of Psychomotor Function: A Randomized Controlled Trial. Adv. Ther..

[4] Song S.W., Kim S., Park J.-H., Cho Y.H., Jeon Y.-G. (2023). Post-induction hypotension with remimazolam versus propofol in patients routinely administered angiotensin axis blockades: A randomized control trial. BMC Anesthesiol..

[5] Kitaura A., Tsukimoto S., Sakamoto H., Hamasaki S., Nakao S., Nakajima Y. (2023). A retrospective comparative study of anesthesia with remimazolam and remifentanil versus dexmedetomidine and remifentanil for transcatheter aortic valve replacement. Sci. Rep..

[6] Curtin F. (2017). Meta-analysis combining parallel and cross-over trials with random effects. Res. Synth. Methods.

[7] Langan D., Higgins J.P., Jackson D., Bowden J., Veroniki A.A., Kontopantelis E., Viechtbauer W., Simmonds M. (2019). A comparison of heterogeneity variance estimators in simulated random-effects meta-analyses. Res. Synth. Methods.

[8] Zhang Y., Akl E.A., Schünemann H.J. (2019). Using systematic reviews in guideline development: The GRADE approach. Res. Synth. Methods.

[9] Doi M., Morita K., Takeda J., Sakamoto A., Yamakage M., Suzuki T. (2020). Efficacy and safety of remimazolam versus propofol for general anesthesia: A multicenter, single-blind, randomized, parallel-group, phase IIb/III trial. J. Anesthesia.

[10] Shi F., Chen Y., Li H., Zhang Y., Zhao T. (2022). Efficacy and Safety of Remimazolam Tosilate versus Propofol for General Anesthesia in Cirrhotic Patients Undergoing Endoscopic Variceal Ligation. Int. J. Gen. Med..

[11] Luo W., Sun M., Wan J., Zhang Z., Huang J., Zhang J., Xiong W., Xia L., Xu P., Miao C. (2023). Efficacy and safety of remimazolam tosilate versus propofol in patients undergoing day surgery: A prospective randomized controlled trial. BMC Anesthesiol..

[12] Oh E.J., Chung Y.J., Lee J.-H., Kwon E.J., Choi E.A., On Y.K., Min J.-J. (2023). Comparison of propofol vs. remimazolam on emergence profiles after general anesthesia: A randomized clinical trial. J. Clin. Anesthesia.

[13] Kim H.-J., Kim J.-Y., Park H.-S., Kim H., Ro Y.-J., Koh W.U. (2024). Effect of Remimazolam- versus Propofol-Based Total Intravenous General Anesthesia on Intraoperative Hemodynamic Stability for Major Spine Surgery in the Prone Position: A Randomized Controlled Trial. Medicina.

[14] Zhang J., Zhang J., Wang Y., Bai X., Guo Q., Liu W., Li H., Zhu F., Wang X., Jiang X. (2024). Effect of remimazolam vs propofol on emergence from general anesthesia in patients undergoing cerebral endovascular procedures: A randomized controlled, non-inferiority trial. J. Clin. Anesthesia.

[15] Lee J., Han D.W., Song Y., Lee J., Jeon S., Kim M.H. (2024). Quality of Postoperative Recovery in Total Intravenous Anesthesia between Remimazolam and Propofol for Intraoperative Neurophysiological Monitoring: A Prospective Double-Blind Randomized Controlled Trial. J. Pers. Med..

[16] Yang J.-J., Lei L., Qiu D., Chen S., Xing L.-K., Zhao J.-W., Mao Y.-Y., Yang J.-J. (2023). Effect of Remimazolam on Postoperative Delirium in Older Adult Patients Undergoing Orthopedic Surgery: A Prospective Randomized Controlled Clinical Trial. Drug Des. Dev. Ther..

[17] Wang L., Wang Y., Ma L., Wang Y., Mu X., Huang Z., Zheng Z., Nie H. (2023). Cardiopulmonary Adverse Events of Remimazolam versus Propofol During Cervical Conization: A Randomized Controlled Trial. Drug Des. Dev. Ther..

[18] Sneyd J.R., Absalom A.R., Barends C.R., Jones J.B. (2022). Hypotension during propofol sedation for colonoscopy: A retrospective exploratory analysis and meta-analysis. Br. J. Anaesth..

[19] Zhu H., Su Z., Zhou H., Lu J., Wang X., Ji Z., Chen S., Wang X., Yao M., Lu Y. (2024). Remimazolam Dosing for Gastroscopy: A Randomized Noninferiority Trial. Anesthesiology.

[20] Zhang S., Wang J., Ran R., Peng Y., Xiao Y. (2022). Efficacy and safety of remimazolam tosylate in hysteroscopy: A randomized, single-blind, parallel controlled trial. J. Clin. Pharm. Ther..

[21] Zhang X., Li S., Liu J. (2021). Efficacy and safety of remimazolam besylate versus propofol during hysteroscopy: Single-centre randomized controlled trial. BMC Anesthesiol..

[22] Li Z., Yuan D., Yu Y., Xu J., Yang W., Chen L., Luo N. (2024). Effect of remimazolam vs propofol in high-risk patients undergoing upper gastrointestinal endoscopy: A non-inferiority randomized controlled trial. Trials.

[23] Yang L., Zhang J., Xiao N., Chen J., Liu H., He X., Xiao X., Zhang F. (2024). Clinical Trial Comparing Remimazolam with Propofol During Intravenous Anesthesia: A Prospective Randomised Clinical Trial. Comb. Chem. High Throughput Screen..

[24] Huang X., Cao H., Zhang C., Lan H., Gong X., Li R., Lin Y., Xu B., Chen H., Guan X. (2023). The difference in mean arterial pressure induced by remimazolam compared to etomidate in the presence of fentanyl at tracheal intubation: A randomized controlled trial. Front. Pharmacol..

