# Supplementary material for: Remimazolam Versus Propofol in General Anesthesia of Complex Surgery in Critical and Non-Critical Patients: Meta-Analysis of Randomized Trials
Source: J Clin Med. 2024 Dec 20;13(24):7791. doi: 10.3390/jcm13247791 (PMC11728358; doi:10.3390/jcm13247791)
Supplement: Supplementary file 1 [file jcm-13-07791-s001.zip › jcm-3304672-supplementary.pdf]

Supplementary files

Supplementary Table S1. Strategy search

Supplementary Table S2. GRADE assessment

Table S1. Strategy search.

SEARCH ENGINE	STRATEGY	RESULTS
PUBMED	#1= (remimazolam OR anerem OR biprazine OR rf10007 OR "cns 7056" OR "ono2745" OR "gw502056x")<br><br>#2= (Propofol OR "2,6-Diisopropylpheno*" OR "2,6-Bis(1-methylethyl)phenol" OR "Disoprofol Diprivan" OR "Disoprivan" OR "Fresofol" OR "ICI-35,868" OR "ICI 35,868" OR "ICI35,868" OR "ICI-35868" OR "ICI 35868" OR "ICI35868" OR "Ivofol" OR "Propofol Fresenius" OR "Propofol MCT" OR "Propofol Rovi" OR "Propofol-Lipuro" OR "Recofol" OR "Aquafol" OR "Propofol Abbott")<br><br>#3= (“Randomized Controlled Trial” OR “Randomised Controlled Trial” OR “Clinical Trials, Randomized" OR “Trials, Randomized Clinical” OR “Controlled Clinical Trials, Randomized” OR “controlled clinical trial” OR “clinical trial”)	121
SCOPUS	TITLE-ABS-KEY(remimazolam OR anerem OR biprazine OR rf10007 OR "cns 7056" OR "ono2745" OR "gw502056x") AND (TITLE-ABS-KEY(Propofol OR "2,6-Diisopropylpheno*" OR "2,6-Bis(1-methylethyl)phenol" OR "Disoprofol Diprivan" OR "Disoprivan" OR "Fresofol" OR "ICI-35,868" OR "ICI 35,868" OR "ICI35,868" OR "ICI-35868" OR "ICI 35868" OR "ICI35868" OR "Ivofol" OR "Propofol Fresenius" OR "Propofol MCT" OR "Propofol Rovi" OR "Propofol-Lipuro" OR "Recofol" OR "Aquafol" OR "Propofol Abbott")) AND (TITLE-ABS-KEY (“Randomized Controlled Trial" OR “Randomised Controlled Trial” OR “Clinical Trials, Randomized" OR “Trials, Randomized Clinical” OR “Controlled Clinical Trials, Randomized” OR “controlled clinical trial” OR “clinical trial”))	191
WEB OF SCIENCE	#1= (remimazolam OR anerem OR biprazine OR rf10007 OR "cns 7056" OR "ono2745" OR "gw502056x")<br><br>#2= (Propofol OR "2,6-Diisopropylpheno*" OR "2,6-Bis(1-methylethyl)phenol" OR "Disoprofol Diprivan" OR "Disoprivan" OR "Fresofol" OR "ICI-35,868" OR "ICI 35,868" OR "ICI35,868" OR "ICI-35868" OR "ICI 35868" OR "ICI35868" OR "Ivofol" OR "Propofol Fresenius" OR "Propofol MCT" OR "Propofol Rovi" OR "Propofol-Lipuro" OR "Recofol" OR "Aquafol" OR "Propofol Abbott")<br><br>#3= (“Randomized Controlled Trial” OR “Randomised Controlled Trial” OR “Clinical Trials, Randomized" OR “Trials, Randomized Clinical” OR “Controlled Clinical Trials, Randomized” OR “controlled clinical trial” OR “clinical trial”)	89
EMBASE	#1= 'remimazolam'/exp OR 'remimazolam'<br>#2= 'propofol'<br>#3= 'randomized controlled trial'	160

Table S2. GRADE assessment.

Outcomes	№ of participants (studies) Follow-up	Certainty of the evidence (GRADE)	Relative effect (95% CI)	Anticipated absolute effects	
				Risk with [Comparación]	Risk difference with [Intervención]
**Intraoperative hypotension assessed with: RR**	**1133 (9 RCTs)**	⊕⊕⊕◯ **Moderate**	**RR 0.62** (0.50 to 0.76)	568 per 1,000	**216 fewer per 1,000** (284 fewer to 136 fewer)

Outcomes	№ of participants (studies) Follow-up	Certainty of the evidence (GRADE)	Relative effect (95% CI)	Anticipated absolute effects	
				Risk with [Comparación]	Risk difference with [Intervención]
Incidence respiratory depression assessed with: RR	290 (3 RCTs)	⨁⨁⨁◯ Moderate	**RR 0.28** (0.09 to 0.82)	110 per 1,000	**79 fewer per 1,000** (100 fewer to 20 fewer)
Bradycardia assessed with: RR	640 (4 RCTs)	⨁⨁⨁◯ Moderate	**RR 0.61** (0.36 to 1.06)	102 per 1,000	**40 fewer per 1,000** (65 fewer to 6 more)
Injection site pain assessed with: RR	664 (4 RCTs)	⨁⨁⨁◯ Moderate	**RR 0.14** (0.02 to 0.94)	116 per 1,000	**100 fewer per 1,000** (114 fewer to 7 fewer)

*__The risk in the intervention group__ (and its 95% confidence interval) is based on the assumed risk in the comparison group and the **relative effect** of the intervention (and its 95% CI).

CI: confidence interval; **RR:** risk ratio

GRADE Working Group grades of evidence

High certainty: we are very confident that the true effect lies close to that of the estimate of the effect.

Moderate certainty: we are moderately confident in the effect estimate: the true effect is likely to be close to the estimate of the effect, but there is a possibility that it is substantially different.

Low certainty: our confidence in the effect estimate is limited: the true effect may be substantially different from the estimate of the effect.

Very low certainty: we have very little confidence in the effect estimate: the true effect is likely to be substantially different from the estimate of effect.
